# Parents’ satisfaction with information received on psychotropic drugs used by adolescents in a Mental Health Unit

**DOI:** 10.1590/1984-0462/2022/40/2021012IN

**Published:** 2022-05-11

**Authors:** Dafny Oliveira de Matos, Patrícia Medeiros-Souza, Renata Passos de Melo, Ricardo Azevedo de Menezes, Noemia Urruth Leão Tavares

**Affiliations:** a Universidade de Brasília, Brasília, DF, Brazil.; b Multiprofessional Residency Program in Family and Community Health, School of Health Sciences, Brasília, DF, Brazil.; c Health Secretariat of the Federal District, Brasília, DF, Brazil.

**Keywords:** Adolescent, Patient satisfaction, Psychotropic drugs, Mental health, Mental disorders, Adolescente, Satisfação do paciente, Psicotrópicos, Saúde mental, Transtornos mentais

## Abstract

**Objective:**

To analyze the satisfaction of those responsible for adolescents with information received for the use of psychotropic drugs.

**Methods:**

Cross-sectional study carried out in a reference outpatient clinic in Brasília between 2017 and 2019. It involved 173 legal representatives of adolescents diagnosed with Mental and Behavioral Disorders using psychotropic drugs. In order to identify the level of satisfaction about the information received on psychotropic drugs, the Satisfaction with Information about Medicines Scale (SIMS) was used.

**Results:**

Most guardians were dissatisfied with the information received on psychotropic drugs (n=112; 64.7%). The dissatisfaction with information about potential problems of medication was the one that stood out the most (n=127; 73.4%) when compared to information about action and usage (n=89; 51.5%). Participants considered information on the impact of medication on the adolescent’s sexual life unsatisfactory or nonexistent.

**Conclusions:**

The parents’ satisfaction with the information received about psychotropic drugs was low. Participants showed dissatisfaction with the information about potential problems, especially related to the impact on the sexual life of their tutored.

## Resumo

**Objetivo:**

Analisar a satisfação dos responsáveis por adolescentes com as informações recebidas para o uso dos medicamentos psicofármacos.

**Métodos:**

Estudo transversal realizado em ambulatório de referência entre 2017 e 2019, em Brasília, com 173 responsáveis por adolescentes diagnosticados com transtornos mentais e comportamentais em uso de psicofármacos. O questionário Satisfaction with Information about Medicines Scale (SIMS) foi utilizado para identificar o grau de satisfação em relação às informações recebidas sobre os medicamentos.

**Resultados:**

A maioria dos responsáveis estava insatisfeita quanto às informações recebidas sobre psicofármacos (n=112; 64,7%). Destacou-se a insatisfação com as informações sobre potenciais problemas do medicamento (n=127; 73,4%), quando comparada à ação e ao uso (n=89; 51,5%). As informações sobre o impacto do medicamento na vida sexual do adolescente foram consideradas insatisfatórias ou inexistentes.

**Conclusões:**

A satisfação dos responsáveis com as informações recebidas sobre psicofármacos foi baixa. Os participantes demonstraram insatisfação com as informações sobre potenciais problemas, especialmente às relacionadas ao impacto na vida sexual dos adolescentes.

## INTRODUCTION

The prevalence of mental disorders in adolescents has increased over the last ten years.^1,2^ Mental health conditions are responsible for the greatest global burden of the disease in adolescents,^1,2^ being considered a public health problem.^3^ Adolescence is a critical period for the development of mental illness, with more than half of the cases diagnosed up to 14 years of age.^2^ Concomitantly, a significant increase was identified in relation to the use of health services for diagnosis and treatment of psychosomatic complaints and mental disorders.^3^ Adherence to treatment is essential for the success of therapy, but there may be some difficulties in this process.^4^

Adherence to pharmacotherapy is multidimensional and involves socioeconomic factors, therapy-related factors, health system and health care team factors, patient-related factors, and condition-related factors.^4^ Low adherence is frequent among people with psychiatric disorders.^5^ The relationship between satisfaction with information received about medications and medication adherence is frequently reported.^5–7^ Dissatisfaction with information received about psychotropic drugs is reported in several studies,^5^ especially with regard to information on adverse reactions and potential problems related to pharmacotherapy.^6,8–11^

Person-centered approaches in the context of drug information are still little explored in science, despite showing good results.^7^ Health professionals tend to overestimate the quantity and quality of information provided to patients.^12^ However, communication between professionals and patients must adjust according to the patients’ needs, with no single model of communication and information exchange.^7,11^ Patient satisfaction with the information received about medications can arise with a more focused communication on what the person wants to know and less in relation to what the professional thinks she should know.^7,13^

The main objective of this study was to analyze the satisfaction profile of those responsible for adolescents regarding the information received about drug treatment for mental disorders. Complementarily, it was meant to identify the types of information on psychotropic drugs that need to be improved by health professionals to increase caregivers’ satisfaction.

## METHOD

A cross-sectional descriptive study carried out in Brasília, Brazil, between August 2017 and January 2019. The target population was composed of parents and/or guardians of adolescents being cared for at a reference clinic in mental health in Brasília, specialized in the care of adolescents and belonging to the Unified Health System (*Sistema Único de Saúde* – SUS), in the Federal District. The clinic has individual or group care by a multidisciplinary team consisting of nurses, pharmacists, occupational therapists, dentists, social workers, nutritionists, psychologists, pediatricians, psychiatrists, and gynecologists. The population assisted by the service is composed of adolescents with symptoms of psychological distress or mild or moderate mental disorders, accompanied by their families or guardians.

The sample size was previously calculated based on the average number of monthly visits performed by a pediatrician or psychiatrist. The estimated population was 580 adolescents, and the estimated sampling error was 5% with a 95% confidence interval (95%CI). The final sample consisted of was 173 pairs of adolescents and their guardians. Inclusion and exclusion criteria were applied to both adolescents and their guardian. Adolescents should be between 10 and 19 years old and be seen at the reference clinic; present diagnoses of mental and behavioral disorders, according to Chapter V of the ICD-10 of the World Health Organization (WHO); and having a prescription for continuous use of medications regulated by Ordinance No. 344/1998,^14^ identified from the medical records of the services. Parents and/or legal guardians must be at least 18 years of age and be legally responsible for the adolescents eligible for the research. Parents who did not understand the questions in the questionnaire were excluded; as well as those who were not present or were absent during the collection or refused to participate.

The sampling of participants was carried out based on the service’s schedule, the adolescents’ medical records were evaluated and all those responsible for adolescents who met the inclusion criteria were invited to participate in the study on the day of the consultation. Data collection occurred through a questionnaire to those responsible for the adolescents. Sociodemographic variables were collected, including gender, education, family income, self-reported skin color, age, and number of dependents of the guardians. Information on adherence to pharmacotherapy by adolescents was also collected with the Medication Adherence Rating Scale (MARS).^15^ The final result of the MARS and its categories “medication adherence behavior”, “attitude toward taking medication” and “negative side effects and attitudes to psychotropic medication” were analyzed as independent variables.^15^

The degree of satisfaction with the information received about medications was assessed by applying the Satisfaction with Information about Medicines Scale (SIMS) questionnaire.^6^ The questionnaire used was translated into Portuguese, from the original instrument, and its testing was performed in pilot phase prior to data collection. The instrument consists of 17 items that outline a profile of satisfaction with information received about drug treatment. Each item refers to a different type of information that must be categorized into one of the five options (too much, about right, too little, none received, none needed). The categorization was made based on the perception of each respondent about the quality of information they received by the multidisciplinary team about each item during the monitoring of their child or dependent in the service. Scores were assigned to each answer.^6,16^

Data obtained after collection were gathered, coded, and analyzed in a database using the Epi Info version 7.0 program. In the descriptive analysis, data were expressed as absolute or relative frequency and measures of central tendency and variability. Data analyses were performed using the IBM SPSS (Statistical Package for the Social Sciences) program, version 23, 2015. The significance level used was 5%.

Categorical variables were associated with satisfactory or unsatisfactory result (SIMS) using Pearson’s chi-square test with continuity correction when necessary (at least one cell with expected frequency less than 5). For nominal variables with only two categories, it was possible to calculate the *odds ratio* and the corresponding 95%CI.

Numerical variables were first evaluated in relation to data distribution using the Kolmogorov-Smirnov test. The null hypothesis of data normality was rejected for the age and number of dependents variables, in addition to the MARS questionnaire result variables. Thus, the nonparametric Mann-Whitney U test was used to assess the association between satisfaction with information about medications and these quantitative variables.

The present research was carried out in accordance with the Resolution of the National Health Council (Conselho Nacional de Saúde – CNS) No. 466/2012.^17^ The project was approved and received by the Research Ethics Committee of *Fundação de Ensino e Pesquisa em Ciências da Saúde* (FEPECS) of the Health Secretariat of the Federal District (SES/DF) on April 26^th^, 2017 at 10:16 am, under Opinion No. 2.050.934. The anonymity of the study participants was maintained and the questionnaires were identified with the letter “R” for those answered by legal guardians (*responsável*), plus the number corresponding to the order in which they were applied.

## RESULTS

The total number of medical records analyzed was 296, of which 123 were excluded because the adolescent was not accompanied by a legal guardian at the time of data collection (n=33); the adolescent was not using a psychotropic drug (n=57); the non-understanding of the questionnaire by the guardian (n=2); absence at the time of the call to answer the questionnaire (n=11); refusal (n=19); and giving up during tool application (n=1). Thus, the final sample consisted of 173 participants.

The most prevalent profile of research participants was female (n=151; 87.28%); with an average age of 44 years, ranging between 29 and 69 years; with education equal to or higher than complete high school (n=110; 63.6%); non-white (n=125; 72.3%); and with less than two minimum wages per family (n=97; 56.7%). The number of children or dependents of each guardian ranged from 0 to 10, with an average of 2.29. Other sociodemographic data are presented in Table 1.

**Table 1.** Sociodemographic data of guardians of adolescents receiving care at a mental health reference clinic in Brasília, Distrito Federal, 2017–2019.

Characteristics	n	SD	Mean
Age (years)	173	8.6	44.0
Number of dependents	173	1.2	2.3
	**n**		**(%)**
Gender			
Female	151		87.3
Male	22		12.7
Education			
At least complete high school	110		63.6
Up to complete high school	63		36.4
Race or color			
White	40		23.1
Non-White	125		72.2
Ignored or undeclared	8		4.6
Family income			
Less than two minimum wages	97		56.7
More than two minimum wages	74		43.3

SD: standard deviation.

Among the pharmacotherapeutic characteristics, the mother was the main responsible for administering medication to adolescents (n=89; 52.0%). The highest frequency of administration was one (n=77; 44.5%) taking of medication per day, followed by two (n=53; 30.6%) and three or more takings (n=43; 24.9%) daily (data not shown in table).

Participants showed low satisfaction with the information received about medications (n=112; 64.7%). The highest degree of satisfaction was with the item “Action and usage” of medication, compared to the satisfaction with information on “Potential problems”. The information “What your medicine is called” received the highest percentage of satisfied participants (n=138; 79.8%), although, at the same time, it was the most classified information as “too much” (n=23; 13.3%). The information received with less satisfaction (n=59; 34.1%) was “Whether the medication will affect your sex life”.

The total and subscale satisfaction profile, in addition to the detailed information profile about the drugs and the individual classification of items, are described in Table 2.

**Table 2.** Satisfaction with information received about medications, “Action and usage” and “Potential problems”, and detailed information profile on medications and individual classification of items.

			Scale	n	Answers indicating dissatisfaction, n (%)			Aggregate dissatisfaction, n (%)	Aggregate satisfaction *, n (%)
					Too much	Too little	None received		
Action and use	89 (51.4)	84 (48.5)	What your medicine is called.	171	23 (13.3)	7 (4.0)	3 (1.7)	33 (19.1)	138 (79.8)
			What your medicine is for.	171	14 (8.1)	16 (9.2)	7 (4.0)	37 (21.4)	134 (77.5)
			What it does.	170	12 (6.9)	34 (19.6)	10 (5.8)	56 (32.4)	114 (65.9)
	Total dissatisfaction. n (%)	Total satisfaction. n (%)	How it works.	172	8 (4.6)	44 (25.4)	16 (9.2)	68 (39.3)	104 (60.1)
			How long it will take to act.	170	8 (4.6)	33 (19.1)	23 (13.3)	64 (37.0)	106 (61.3)
			How you can tell if it is working.	172	6 (3.5)	37 (21.4)	14 (8.1)	57 (32.9)	115 (66.5)
			How long you will need to be on your medicine.	171	15 (8.7)	41 (23.7)	37 (21.4)	93 (53.8)	78 (45.1)
			How to use your medicine.	171	21 (12.1)	9 (5.2)	6 (3.5)	36 (20.8)	135 (78.0)
			How to get a further supply.	171	17 (9.8)	16 (9.2)	10 (5.8)	43 (24.8)	128 (74.0)
Potential problems	127 (73.4)	46 (26.6)	Whether the medicine has any unwanted effects (side effects).	171	4 (2.3)	37 (21.4)	36 (20.8)	77 (44.5)	94 (54.3)
			What are the risks of you getting side effects.	170	7 (4.0)	50 (28.9)	47 (27.2)	104 (60.1)	66 (38.1)
			What you should do if you experience unwanted side effects.	165	3 (1.7)	48 (27.9)	48 (27.9)	99 (57.6)	66 (38.4)
	Total dissatisfaction. n (%)	Total satisfaction. n (%)	Whether you can drink alcohol while using the medicine.	171	16 (9.2)	19 (11.0)	36 (20.8)	71 (41.0)	100 (57.8)
			Whether the medicine interferes with other medicines.	172	6 (3.5)	48 (27.7)	50 (28.9)	104 (60.1)	68 (39.3)
			Whether the medication will make you feel drowsy.	170	10 (5.8)	32 (18.5)	26 (15.0)	68 (39.3)	102 (59.0)
			Whether the medication will affect your sex life.	170	4 (2.3)	54 (31.2)	53 (30.6)	111 (64.2)	59 (34.1)
			What you should do if you forget to take a dose.	172	8 (4.6)	39 (22.5)	30 (17.3)	77 (44.5)	95 (54.9)
Total	112 (64.7)	61 (35.3)							

*The aggregated satisfaction corresponds to the answers that indicate satisfaction, being they “about right” or “none needed”.

Among the statistical tests performed, Table 3 shows that there was no statistically significant association between the variables gender, race, income, and satisfaction with information about medications. There was a significant difference in terms of education and satisfaction in the “Action and usage” and “Potential problems” of medication subscales. It is observed that guardians who had completed high school were 2.24 (95%CI 1.18–4.24) times more likely to be satisfied with the information about action and usage and 2.30 (95%CI 1 .07–4.93) with information about potential problems compared to guardians who had incomplete high school education. In relation to total satisfaction, there was no significant difference.

**Table 3.** Analysis of the association of nominal and ordinal qualitative variables, and the result of the Satisfaction with Information about Medicines Scale questionnaire applied to those responsible for adolescents receiving care at a reference mental health clinic in Brasília, Distrito Federal, 2017–2019.

			Action and use			p-value*	OR	Potential problems			p-value*	OR	Total satisfaction			p-value*	OR
			Satisfied	Dissatisfied	Total			Satisfied	Dissatisfied	Total			Satisfied	Dissatisfied	Total		
Gender	Female	n	74	77	151	0.267	1.68	43	108	151	0.310	1.79	53	96	149	0.445	1.47
		%	49.0	51.0	100.0			28.5	71.5	100.0			35.6	64.4	100.0		
	Male	n	8	14	22			4	18	22			6	16	22		
		%	36.4	63.6	100.0			18.2	81.8	100.0			27.3	72.7	100.0		
Race	White	n	19	21	40	0.886	0.95	12	28	40	0.807	1.10	16	24	40	0.564	1.24
		%	47.5	52.5	100.0			30.0	70.0	100.0			40.0	60.0	100.0	
	Non-White	n	61	64	125			35	90	125			43	80	123	
		%	48.8	51.2	100.0			28.0	72.0	100.0			35.0	65.0	100.0	
Income	Less than two minimum wages	n	40	57	97	0.066		24	73	97	0.358		27	69	96	0.052	
		%	41.2	58.8	100.0		0.56	24.7	75.3	100.0		0.73	28.1	71.9	100.0		0.53
	More than two minimum wages	n	41	33	74			23	51	74			31	42	73		
		%	55.4	44.6	100.0			31.1	68.9	100.0			42.5	57.5	100.0		
Education	At least complete high school		60	50	110	**0.013**		36	74	110	**0.030**		43	66	109	0.071	
			54.5	45.4	100.0		2.24	32.7	67.3	100.0		2.30	39.4	60.5	100.0		1.87
	Up to incomplete high school	n	22	41	63			11	52	63			16	46	62		
		%	34.9	65.1	100.0			17.5	82.5	100.0			25.8	74.2	100.0		
Total		n	82	91	173			47	126	173			59	112	171		
		%	47.4	52.6	100.0			27.2	72.8	100.0			34.5	65.5	100.0		

OR: odds ratio; * Pearson’s chi-square test with continuity correction when necessary.

**Figure 1.**
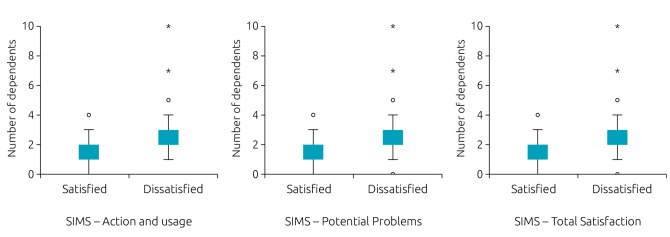
Box plot of the association between the number of dependents and the result of the Satisfaction with Information about Medicines Scale questionnaire applied to those responsible for adolescents receiving care at a reference mental health clinic in Brasília, Distrito Federal, 2017–2019.

For quantitative variables, the number of dependents was statistically associated with satisfaction with information about medications both in the total score and in the subscales “Action and usage” and “Potential problems” (Table 4). It is observed that guardians who were dissatisfied with information about medications had significantly more dependents. This difference is best evidenced in Figure1.

**Table 4.** Analysis of the association of quantitative variables and the result of the Satisfaction with Information about Medicines Scale questionnaire applied to those responsible for adolescents receiving care at a reference mental health clinic in Brasília, Distrito Federal, 2017–2019.

	SIMS — action and use				p-value*	SIMS — potential problems				p-value	SIMS total				p-value
	Satisfied		Dissatisfied			Satisfied		Dissatisfied			Satisfied		Dissatisfied		
	Md	IQ	Md	IQ		Md	IQ	Md	IQ		Md	IQ	Md	IQ	
Age	41.0	11.0	43.0	11.0	0.426	41.0	12.0	43.0	10.0	0.977	41.0	11.0	43.0	11.0	0.552
Number of dependents	2.0	1.0	2.0	1.0	0.001	2.0	1.0	2.0	1.0	0.035	2.0	1.0	2.0	1.0	0.011
MARS — *medication adherence behavior*	2.0	2.0	2.0	2.0	0.141	2.0	2.0	2.0	2.0	0.878	2.0	2.0	2.0	2.0	0.773
MARS — *attitude toward taking medication*	2.5	1.0	3.0	1.0	0.818	3.0	2.0	2.0	1.0	0.065	3.0	1.0	2.0	1.0	0.438
MARS — *negative side effects and attitudes to psychotropic medication*	1.0	1.0	1.0	1.0	0.707	1.0	1.0	1.0	1.0	0.747	1.0	1.0	1.0	1.7	0.486
MARS total	6.0	3.0	6.0	3.0	0.294	6.0	3.0	6.0	3.0	0.334	6.0	3.0	6.0	3.0	0.527

SIMS: Satisfaction with Information about Medicines Scale; Md: median; IQ: interquartile range; MARS: Medication Adherence Rating Scale; *Mann-Whitney U test.

SIMS: *Satisfaction with Information about Medicines Scale*.

## DISCUSSION

This study is a pioneer in exploring the satisfaction profile of guardians of adolescents with the information received by the assistant health teams about the psychotropic drugs used in the treatment of mental disorders.

The participants’ general dissatisfaction with the information explored in this study is remarkable. Participants were more dissatisfied with the information received about potential drug problems when compared to action and usage. These results follow the same pattern found in the literature when they used the SIMS questionnaire for different classes of medications.^8–10,18–21^ Klewitz et al.^8^ researched the necessary information about immunosuppressants for 440 kidney transplant patients over 15 years of age and found that 46.1% of respondents were dissatisfied with the information in the item “Potential problems” and 26.7% with the item “Action and usage”. A similar pattern of results was found in people with bipolar disorder,^22^ systemic lupus erythematosus,^18^ using antineoplastic agents^19,20^ or antiretrovirals^16^ and other conditions.^9,10^ People often do not take their medications due to ignorance regarding its purposes or to lack of information, especially about potential drug-related problems,^18,19^ as seen in the current study. The perception of professionals about the quality of the information they provide is often at variance with the perception of patients or their caregivers.^11^

A cross-sectional study^21^ conducted in London with 1,400 people with heart problems and health professionals showed that health professionals commonly advise on the action and usage of medications and not on potential problems. In this study by Auyeung,^21^ physicians and nurses understand that it is not their responsibility to guide patients about potential problems in pharmacotherapy, as is a responsibility assigned to pharmacists.^21^ In the same study, pharmacists agree that it is their responsibility to advise on potential problems and, in the item “Action and usage”, on “How long it will take to act”.^21^ These results highlight the importance of the multidisciplinary team in approaching the use of medications.

The information with the greatest dissatisfaction identified, regardless of the subscale, was regarding the interference of the medication in adolescents’ sexual life. Similar patterns of dissatisfaction with the information on the item “Whether the medication will affect your sex life” are found in the literature in investigations with adults,^8,9,20,21^ but they do not address the use of medication and its interference in the sexual health of teenagers. Sexuality is undeniably a significant part of the adolescent experience.^2^ However, health professionals seem to be unprepared to discuss and provide guidance on this subject, although they understand that the approach to the theme must be holistic and person-centered.^23,24^

Martins et al.^25^ conducted a workshop with five young people aged between 16 and 24 who had cancer. These participants reported that they had many doubts about the impact of their pharmacotherapies on their sex life, but there was little space to talk about it. When it occurred, it was for a short time and with the health professional in visible discomfort.^25^ These young people reported that they feel embarrassed to start the conversation and that health professionals do not initiate it either.^25^ Furthermore, they would like to be open to talk on the subject throughout the therapeutic follow-up, with trained professionals, in appropriate spaces, without the presence of parents.^25^ They also stated that it would be important to be able to discuss how to manage the adverse effects of medications.^25^ In the context of psychotropic drugs, adverse reactions reported are decreased sexual desire and sexual dysfunction,^24^ as well as gynecomastia, hyperprolactinemia, and diarrhea,^26^ which can affect self-esteem and sexual identity,^25,26^ in addition to being a motivator for treatment interruption.^27^

In addition, it is important to consider that there are interactions that are still not so clarified in the literature between hormonal contraceptives and some psychotropic drugs.^28^ The high prevalence of chronic drug use in female adolescents, especially oral contraceptives, is described nationally,^29^ which reinforces the importance of orienting adolescents about the impacts of their medications on their sexual lives.

Participants also showed great dissatisfaction with the items “What are the risks of you getting side effects” and “Whether the medicine interferes with other medicines”. These results are consistent with those found in the literature.^8,10,20,21,30^ Kusch et al.^30^, based on a scope review with 12 studies published between 2000 and 2017, described what information patients would like to receive. In this review, information related to safety, especially regarding adverse effects and drug interactions, were the most required among the studies evaluated.^30^ Concerns or beliefs regarding adverse effects can negatively interfere with medication adherence, even promoting intentional non-adherence.^19^ Zhang et al.,^18^ in a cross-sectional study with 210 patients with systemic lupus erythematosus conducted in China, revealed that talking about adverse effects can reduce feelings of anxiety caused by lack of knowledge about the effects of the drug and improve the quality of life of people who use them. Therefore, informing about adverse effects can reduce the chances of intentional non-adherence.^10^ The belief of health professionals that informing about adverse effects or drug interactions could harm the proposed treatment is not supported by the literature.^11,30^

Despite the fact that there was a statistically positive relationship between the number of addicts and satisfaction with the information received about psychotropic drugs, further studies are needed to understand this possible association.

As limitations, this research considered the use of psychotropic drugs without segmenting clinical conditions or type of medication with the intention of increasing the external validity of the investigation. The exclusion of guardians who did not understand the questionnaire may overestimate the satisfaction results. The language barrier in relation to the validated tool may also have been a limiting factor, and to minimize this aspect, guidelines for translation and cultural adaptation were used. There may be a potential bias by participants who responded to questionnaires with socially accepted responses, despite having been informed about the confidentiality of the information.

There is evidence in the international literature that suggests that people are dissatisfied with the information received about medications, especially in the item “Potential problems”. Despite the scarcity of Brazilian studies with the adolescent population and with mental disorders, this study showed similar trends that can be used to address such issues with this population.

The data obtained in this research may serve as drivers to improve the quality of the information provided. Studies that show the degree of satisfaction of guardians and adolescents who receive psychotropic drugs for various clinical conditions are scarce. This study showed low satisfaction of the adolescents’ parents and/or guardians with the information received about medications. Thus, additional studies are needed, with a larger number of adolescents and guardians, which also assess the satisfaction of adolescents and compare groups that received information about medications with others that did not, in order to identify the main barriers to adherence and to implement measures to improve the degree of satisfaction.

It is concluded that satisfaction with the information received about medication by guardians of adolescents with mental disorders is low. In addition, information about potential problems related to pharmacotherapy is still unsatisfactory, especially regarding the impact of the use of medications on adolescents’ sexual life, drug interactions and adverse reactions of pharmacotherapy.
